# Intra-ejaculate sperm selection in female zebra finches

**DOI:** 10.1098/rsbl.2016.0220

**Published:** 2016-06

**Authors:** N. Hemmings, C. Bennison, T. R. Birkhead

**Affiliations:** Department of Animal and Plant Sciences, University of Sheffield, Alfred Denny Building, Western Bank, Sheffield, UK

**Keywords:** sperm sub-populations, sperm morphology, sperm swimming velocity, fertilization

## Abstract

Among internal fertilizers, typically fewer than 1% sperm survive the journey through the oviduct. Several studies suggest that the sperm reaching the ovum—the ‘fertilizing set’—comprise a non-random sub-population, but the characteristics of this group remain unclear. We tested whether oviductal selection in birds results in a morphologically distinct subset of sperm, by exploiting the fact that the fertilizing set are trapped by the perivitelline layer of the ovum. We show that these sperm have remarkably low morphological variation, as well as smaller head size and greater tail length, compared with those inseminated. Our study shows that the morphological composition of sperm—rather than length alone—influences success in reaching the ovum.

## 1. Background

Only a tiny proportion of inseminated sperm reach the site of fertilization in internal fertilizers [1]. This reduction in sperm numbers is assumed to result from oviductal selection [2], ensuring only the ‘fittest’ sperm fertilize [3]. Morphologically abnormal sperm are unable to traverse the oviduct in mammals and birds [4–6], but this alone cannot account for the substantial reduction in numbers, suggesting that morphologically normal sperm are subjected to other subtle forms of selection.

Considerable intra-ejaculate variation exists in sperm traits, and several *in vitro* studies have identified sub-populations of phenotypically distinct sperm within ejaculates (e.g. [7–10]). However, few studies have shown specific sperm sub-populations to be more likely to reach the ovum *in vivo*, so the biological relevance of *in vitro* sperm sub-populations is unclear [11].

Evidence for sperm sub-populations *in vivo* is limited. Studies on rabbits (*Oryctolagus cuniculus*) demonstrated that ‘selected’ sperm, retrieved from the upper oviduct, outcompeted freshly ejaculated (non-selected) sperm upon re-insemination [12,13]. However, the ‘superior’ traits of these sperm were not determined, and a later attempt to replicate this result failed [14]. Cohen & Tyler [15] found that sperm reaching the upper oviduct comprised a ‘non-antigenic’ population, but it was not established whether this was a distinct sub-population or simply a threshold number protected from immune attack.

Studying sperm selection *in vivo* is limited by the technical difficulty of locating the ‘fertilizing set’ [16]; by the time sperm reach the site of fertilization, they are scarce and not easily characterized [17]. Most studies of sperm sub-populations have focused on mammals, but birds provide a more convenient study system because the fertilizing set is comparatively large and is trapped by the outer perivitelline layer (PVL) after fertilization [18]. Analysis of PVL sperm provides a unique, non-invasive method to test the hypothesis that non-random subsets of sperm exist within ejaculates.

Bennison *et al*. [19] recently demonstrated an *in vivo* advantage for long sperm at the inter-male level, but it is not known whether this is reflected at the intra-male level, where sperm morphology is less variable [20]. Here, we use the same species to test whether sperm reaching the ovum represent a morphologically distinct subset of those inseminated by a single male.

## 2. Material and methods

Thirty pairs of male and female zebra finches (*Taeniopygia guttata*) from a large captive population [20], all more than 12 months old and hatched in the same year, were paired in cages (dimensions 0.6 × 0.5 × 0.4 m) with a nest-box and *ad libitum* food, water, cuttlebone and grit.

Sperm were obtained from male faecal samples collected during the mating period (see the electronic supplementary material for validation). Ten morphologically normal sperm per male were imaged (five sperm has previously been shown to provide a representative sample in this species [20]) at 400× magnification using darkfield microscopy (Leica DMBL with Infinity 3 camera, Luminera Corporation). Head, midpiece and tail (i.e. the extension of the flagellum beyond the midpiece) length were measured to 0.01 µm using ImageJ [21], by N.H. with high repeatability (*r* > 0.96 for all traits).

Nest-boxes were checked daily and the first egg was removed for PVL examination as described in [22]. All sperm within a 5 mm radius of the germinal disc were photographed and measured as described above. Eggs with less than 10 PVL sperm (three pairs) were excluded, leaving 27 pairs (10–43 PVL sperm).

Morphological traits of sperm from faecal (unselected) and PVL (selected) samples were compared using linear mixed models (*lmer* function, *lme4* package, R v. 3.1.2 [23]) with trait measurement as the response, sample type (faecal/PVL) as the explanatory variable and male identity as a random factor. Log-transformed coefficients of variation were also compared via paired *t*-tests, to assess whether variance differed between samples. Multiple comparisons remained robust to conservative Bonferroni corrections (*α* = 0.0083 (0.05/6)), with the exception of absolute tail length, which became marginally non-significant (see Results and table 1). *P*-values and effect sizes are reported for each comparison in table 1.

**Table 1.** Comparison of sperm morphology in faecal (non-selected) and PVL (selected) samples. Bold type indicates sperm traits that were significantly different in the PVL subpopulations compared with the faecal population.

sperm morphological trait	faecal mean (±s.d.)^a,b^	PVL mean (±s.d.)^a,c^	estimated effect	*t*-value	*p*-value^d^
total sperm length (µm)	67.10 (±4.95)	67.60 (±4.42)	0.209	1.46	0.145
**tail length (µm)**	**28.08** (**±8.82)**	**28.63** (**±5.59)**	**0**.**438**	**2**.**42**	**0**.**016**
midpiece length (µm)	28.32 (±6.02)	28.50 (±3.36)	0.025	0.15	0.880
**head length (µm)**	**10.70** (**±0.76)**	**10.49** (**±0.64)**	**−0**.**228**	**−9**.**26**	**<0**.**001**
**head/total sperm length**	**0.16** (**±0.02)**	**0.16** (**±0.01)**	**−0**.**004**	**−8**.**82**	**<0**.**001**
midpiece/total sperm length	0.43 (±0.10)	0.42 (±0.06)	**−**0.003	**−**1.05	0.293

^a^Values presented are the grand mean and s.d. calculated across individual male means. The potential effect of male identity was controlled for within the statistical analysis.

^b^*N* = 10 sperm per male in all samples, from 27 males in total.

^c^*N* = 10 or more sperm per male (up to a maximum of 43 sperm), from 27 males in total.

^d^Applying a conservative Bonferroni correction for multiple comparisons sets the significance level at 0.0083 (0.05/6). The significance of tail length is therefore marginal (see main text).

## 3. Results

Overall, there was no significant difference in total sperm length in unselected and selected samples, but selected sperm had shorter heads, a tendency towards longer tails (marginally non-significant when a Bonferroni correction for multiple comparisons was applied), and shorter heads relative to total length (table 1).

**Figure 1.**
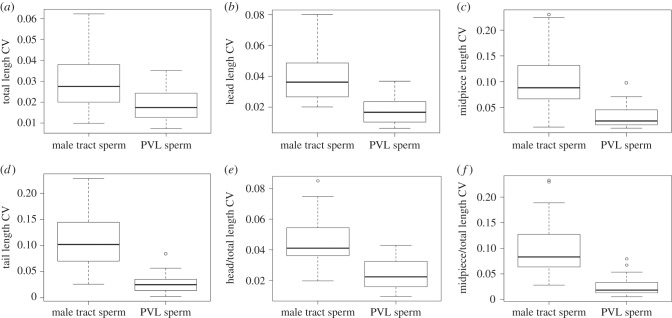
Differences in the coefficients of variation (CV) of sperm traits in male tract sperm (unselected) and PVL sperm (selected) samples: (*a*) total length (mean difference = 0.44 (95% CI: 0.24, 0.64), *t* = 4.55, d.f. = 26, *p* < 0.001); (*b*) head length (mean difference = 0.82 (95% CI: 0.66, 0.98), *t* = 10.30, d.f. = 26, *p* < 0.001); (*c*) midpiece length (mean difference = 1.17 (95% CI: 0.91, 1.43), *t* = 9.26, d.f. = 26, *p* < 0.001); (*d*) tail length (mean difference = 1.49 (95% CI: 1.17, 1.81), *t* = 9.54, d.f. = 26, *p* < 0.001); (*e*) head/total length (mean difference = 0.69 (95% CI: 0.54, 0.83), *t* = 9.78, d.f. = 26, *p* < 0.001); (*f*) midpiece/total length (mean difference = 1.44 (95% CI: 1.23, 1.66), *t* = 14.01, d.f. = 26, *p* < 0.001). Box and whisker plots display the median (horizontal line), interquartile range (box) and full range (whiskers) of the data. Open circles represent outliers, defined as more or less than 1.5 times the upper or lower quartiles, respectively.

As expected, oviductal selection resulted in a decrease in the coefficient of variation in selected compared with non-selected samples for all morphological traits (figure 1).

## 4. Discussion

Sperm reaching the zebra finch ovum were found to be a morphologically non-random subset of those inseminated, characterized by low morphological variation, shorter heads and marginally longer tails.

Previous work in this species has shown a competitive advantage for males producing long sperm [19] (although Bennison *et al*. (C Bennison, N Hemmings, L Brookes, J Slate & TR Birkhead 2016, unpublished data) recently showed that the very longest sperm have reduced swimming velocity). We therefore expected the fertilizing set to comprise relatively long sperm. However, selected sperm were actually characterized by a specific combination of morphological traits: sperm reaching ova tended to have longer tails, shorter heads, and while midpiece length did not differ on average between samples, it (as with all other traits) was significantly less variable in selected sperm.

That variance in sperm morphology might be reduced by oviductal selection is consistent with the results of Immler *et al*. [24], who found reduced intraspecific sperm length variation with increasing sperm competition in a comparative study of passerines. Our findings provide a potential mechanism by which this may evolve: inside the oviduct, sperm with abnormal or extreme morphologies are less able to progress, leaving only a subset with the requisite morphology to traverse the oviduct. When sperm competition risk is high, males are selected to produce uniform sperm so that greater numbers enter the fertilizing set, increasing the likelihood of fertilization [25].

Intra-male variation in sperm morphology is relatively low in the zebra finch [20], but other passerines with higher levels of sperm competition produce sperm of even greater uniformity [24]. In these species, there is presumably less scope for selection to occur within the female tract, owing to the low morphological variation in sperm—compared with the zebra finch, the sub-population of sperm reaching the egg in these species may be less morphologically distinct from the population inseminated. Post-copulatory selection for sperm morphology (by the female) is likely to be stronger when pre-copulatory selection for production of superior sperm (by the male) is weak. Indeed, in species with high levels of sperm competition, sperm production itself appears to be a more discriminatory process [26].

The most variable trait in our unselected samples was midpiece length (figure 1*c*) and this showed the greatest reduction in variance following selection. A curious relationship exists between midpiece and tail length in the zebra finch: the association is generally positive, but sperm with the longest tails tend to have relatively short midpieces [20]. The midpiece comprises a single fused mitochondrion, which has traditionally been considered vital for sperm energetics. It therefore seems unsurprising that sperm with particularly short midpieces and long tails swim less efficiently. However, recent work has revealed a surprising inverse relationship between midpiece length and ATP content in this species (C Bennison, N Hemmings, L Brookes, J Slate & TR Birkhead 2016, unpublished data), raising questions about midpiece function. The ‘optimal’ sperm phenotype here appeared to comprise a midpiece and tail (the rest of the flagellum) of similar length (electronic supplementary material, figure S1); sperm with particularly long midpieces and short tails (or vice versa) rarely reached ova. Longer midpieces may confer greater stability to sperm, reducing the degree to which forward swimming propulsion is inhibited by tail oscillation [27].

Sperm motility is also impeded by drag, which is influenced by head size [28]. Here, sperm in the fertilizing set had relatively short heads, so presumably experienced less viscous resistance (shorter heads have less surface area in contact with the medium they are moving through, and therefore are subjected to less linear drag [29]). Combined with greater thrust from a longer tail, this should promote higher velocity. We suggest that the fertilizing set is morphologically suited for rapid progression through the vagina: sperm with particular morphological traits swim faster through the vagina and have a greater chance of reaching the sperm storage tubules. Selection is likely to occur during the early stages of sperm transport, since high velocity minimizes the risk of immune attack in the vagina [18].

Our results provide evidence that sperm are selected within the zebra finch oviduct, based on morphological traits. Extreme morphologies are removed in favour of a phenotype that promotes swimming efficiency. Successful sperm are not simply the longest, but those that exhibit a specific combination of morphological traits, lending empirical support to the idea that swimming speed and fertilization success are determined by parameters more complex than total sperm length alone.

## Supplementary Material

Supplementary Material

## Data accessibility

Data available from the Dryad digital repository: http://dx.doi.org/10.5061/dryad.vc7ss.
