# Correction to ‘Intra-ejaculate sperm selection in female zebra finches’

**DOI:** 10.1098/rsbl.2018.0004

**Published:** 2018-02-28

**Authors:** N. Hemmings, C. Bennison, T. R. Birkhead

*Biol. Lett.* 12, 20160220. (Published online 8 June 2016). (doi:10.1098/rsbl.2016.0220)

Owing to an error in one of the datasets, a small subset of the total sperm length data used in the original analyses of the paper ‘Intra-ejaculate sperm selection in female zebra finches’ was incorrect (the lengths of the sperm components had been incorrectly summed). The file has now been replaced with a corrected version on Dryad, and we have re-analysed the relevant data. None of the results or overall conclusions of the paper have changed, but the estimates and *p*-values obtained from the analyses are slightly different to those originally reported, as follows:

The difference between total sperm length in faecal and perivitelline layer samples:

original analysis: estimated effect = 0.209, *t* = 1.46, *p* = 0.145

*corrected analysis: estimated effect* = 0.234, *t* = 1.64, *p* = 0.100.

The difference between head length relative to total sperm length in faecal and perivitelline layer samples:

original analysis: estimated effect = −0.004, *t* = −8.82, *p* < 0.001

*corrected analysis: estimated effect* = −0.004, *t* = −8.92, *p* < 0.001.

The difference between midpiece length relative to total sperm length in faecal and perivitelline layer samples:

original analysis: estimated effect = −0.003, *t* = −1.05, *p* = 0.293

*corrected analysis: estimated effect* = −0.003, *t* = −1.14, *p* = 0.255.

The difference between the coefficients of variation of total sperm length in faecal and perivitelline layer samples:

original analysis: *t* = 4.55, d.f. = 26, *p* < 0.001

*corrected analysis*: *t* = 4.78, d.f. = 26, *p* < 0.001.

The difference between the coefficients of variation of head length relative to total sperm length in faecal and perivitelline layer samples:

original analysis: *t* = 9.78, d.f. = 26, *p* < 0.001

*corrected analysis*: *t* = 9.84, d.f. = 26, *p* < 0.001.

The difference between the coefficients of variation of midpiece length relative to total sperm length in faecal and perivitelline layer samples:

original analysis: *t* = 14.01, d.f. = 26, *p* < 0.001

*corrected analysis*: *t* = 13.74, d.f. = 26, *p* < 0.001.

