# Supplementary material for: Intra-ejaculate sperm selection in female zebra finches
Source: Biol Lett. 2016 Jun;12(6):20160220. doi: 10.1098/rsbl.2016.0220 (PMC4938051; doi:10.1098/rsbl.2016.0220)
Supplement: Supplementary Material [file rsbl20160220supp1.docx]

ESM1: Using faecal sample sperm to represent the unselected ejaculate population: Methods and validation.

Male birds in breeding condition continuously discharge sperm (passively) from the vas deferens into the cloaca and these are voided with faeces. Immler & Birkhead [1] showed that faecal samples provide a non-invasive method of obtaining sperm samples for morphological analysis, and sperm from faecal samples are morphologically indistinguishable from ejaculated sperm. Therefore, one day after pairing, a faecal sample was collected from each male as a non-selected sperm sample for morphological measurement. We verified Immler & Birkhead’s [1] results by comparing faecal sperm with those taken from the seminal glomera of 13 males that were used for a separate study, and found no significant differences in morphological traits (head, midpiece and tail length) of sperm from faecal and seminal glomera samples, both in terms of absolute measures and variation (Table S1). We therefore assumed faecal sperm to be representative of the unselected ejaculate sperm population.

To avoid handling-related stress, faecal samples were collected from males by inserting a non-absorbent sheet at the bottom of each cage, which was removed once the male had defecated. The sample was stored in 5% formalin before analysis. Ten morphologically normal sperm per male were photographed at 400x magnification using darkfield microscopy (Leica DMBL with an Infinity 3 camera, Luminera Corporation). Morphological traits –head, midpiece and tail length [2] – were measured to 0.01µm using ImageJ [3]. All measurements were taken by the same person (NH) with high repeatability (r > 0.96 for all traits).

ESM2: Table S1. Comparisons of morphological traits of sperm from faecal and seminal glomera samples

| sperm morphological trait |  |  |  |  |
| --- | --- | --- | --- | --- |
|  |  |  |  |  |
| *absolute measures^1^* | faecal mean (± s.d.)^3,4^ | seminal glomera mean (± s.d.)^3,4^ | t | p |
|  |  |  |  |  |
| head length | 10.19 (± 0.79) | 10.27 (± 0.79) | 0.62 | 0.533 |
| midpiece length | 30.59 (± 4.69) | 30.58 (± 4.65) | -0.01 | 0.989 |
| tail length | 20.26 (± 6.19) | 20.15 (± 6.15) | -0.18 | 0.857 |
| total length | 61.04 (± 6.19) | 61.00 (± 6.19) | -0.07 | 0.944 |
|  |  |  |  |  |
| *coefficients of variation^2^* |  |  | V | p |
| head length | 0.064 (± 0.034) | 0.062 (± 0.037) | 49 | 0.839 |
| midpiece length | 0.091 (± 0.043) | 0.086 (± 0.041) | 64 | 0.216 |
| tail length | 0.155 (± 0.093) | 0.155 (± 0.088) | 50 | 0.790 |
| total length | 0.053 (± 0.034) | 0.052 (± 0.033) | 53 | 0.636 |
|  |  |  |  |  |

^1^Difference between faecal and seminal glomera sperm analysed using a generalised linear mixed model for each trait, with sample type as the explanatory variable and male identity as a random effect

^2^Coefficients of variation for both samples per male were analysed on a pairwise basis using a paired Wilcoxon test for each morphological trait

^3^Values presented are the grand mean and s.d. calculated across individual male means. The potential effect of male identity was controlled for within the statistical analysis.

^4^N = 10 sperm in all samples, from 13 males in total.

ESM3: Figure S1. Ratios of midpiece to tail length in unselected and selected sperm samples. Box and whisker plots are as in Figure 1 (main text).

References

[1] Immler, S. & Birkhead, T.R. 2005. A non-invasive method for obtaining spermatozoa from birds. *Ibis* **147**: 827-830.

[2] Birkhead, T.R., Burke, T. Zann, R., Hunter, F.M. & Krupa, A.P. 2005. Genetic effects on sperm design in the zebra finch. *Nature* **434**: 383-387.

[3] Schneider, C.A., Rasband, W.S. & Eliceiri, K.W. 2012. NIH Image to ImageJ: 25 years of image analysis. *Nat. Methods* **9**: 671-675.
